# Gastric metastasis and transformation of primary lung adenocarcinoma to small cell cancer after acquired resistance to epidermal growth factor receptor tyrosine kinase inhibitors: A case report

**DOI:** 10.1097/MD.0000000000027289

**Published:** 2021-10-01

**Authors:** Jing Liu, Lei Xia, Yuan Peng, Yu Sheng Huang, Zhen Zhou Yang

**Affiliations:** Department of Oncology, the Second Affiliated Hospital of Chongqing Medical University, Chongqing, China.

**Keywords:** case report, gastric metastasis, neoadjuvant/adjuvant epidermal growth factor receptor tyrosine kinase inhibitors treatment, small cell lung cancer, transformation

## Abstract

**Rationale:**

Transformation to small cell lung cancer (SCLC) is one of the mechanisms of resistance to epidermal growth factor receptor tyrosine kinase inhibitors (EGFR-TKIs). However, no standard treatment is available after the transformation. In addition, gastric metastasis of primary lung cancer is rarely observed; thus, little is known about its metastatic characteristics.

**Patient concerns:**

A 58-year-old male patient was treated with gefitinib (0.25 g /day) as the 1st line treatment due of recurrence after surgical resection for EGFR exon 19 mutation pulmonary adenocarcinoma. However, he experienced recurrence with positive T790 M, and osimertinib (80 mg/day) was administered as the 2nd line therapy.

**Diagnosis:**

One year and 6 months after osimertinib initiation, he complained of stomachache, and a diagnostic gastroscopy biopsy confirmed small cell lung cancer in the gastric body, indicating osimertinib-induced phenotypic transformation.

**Interventions and outcomes:**

The patient was treated with etoposide and platinum chemotherapy and maintenance therapy with osimertinib. Finally, the patient achieved a partial response after 4 cycles.

**Lessons:**

Timely second biopsies should be considered in the diagnosis of phenotypic transformation. After transformation, chemotherapeutic treatment with etoposide and platinum and maintenance therapy with osimertinib inhibited the progression of the disease.

## 1 Introduction

The use of epidermal growth factor receptor tyrosine kinase inhibitors (EGFR-TKIs) has been approved as a treatment for advanced non-small cell lung carcinoma (NSCLC) with EGFR-activating mutations. However, almost all patients treated with EGFR-TKIs eventually develop resistance to these agents. The T790 M mutation plays an important role in acquired resistance to treatment with first- or second-generation EGFR-TKIs. If an NSCLC patient acquires a T790 M mutation, the third-generation EGFR-TKI osimertinib is recommended.^[1]^ This treatment induced a remarkable response compared with standard cytotoxic chemotherapy in patients with EGFR-positive NSCLC.^[2–3]^ However, despite this favorable response, these patients experience disease progression after an average time of approximately 12 months.^[4]^ Several resistance mechanisms have been identified, including EGFR C797S mutation, EGFR MET or HER2 amplification, phosphoinositide 3- kinase pathway activation, and rare transformation from lung adenocarcinoma (LUAD) to small cell lung cancer (SCLC).^[5–8]^ Transformation to SCLC is considered a mechanism of resistance to EGFR-TKI treatment in approximately 5% of patients.^[9]^

Patients with stage IIIA-N2 NSCLC harbor considerable heterogeneity with variable ipsilateral mediastinal lymph node involvement (Robinson classification IIIA1 to IIIA4).^[10]^ The treatment includes the following options: surgery followed by adjuvant chemotherapy, neoadjuvant therapy followed by surgical resection, or definitive chemoradiation. Downstaging of N2 disease with neoadjuvant chemotherapy is associated with an approximately 70% resectability rate.^[11]^ However, EGFR-TKIs significantly prolong the progression-free survival of patients with advanced NSCLC who are positive for EGFR mutations compared with chemotherapy as first-line treatment.^[12–16]^ The potential efficacy of neoadjuvant EGFR-TKI therapy in patients with resectable NSCLC has been described in case reports and small-scale nonrandomized clinical trials.^[17–20]^ Zhong et al recently described the results of a randomized phase II EMERGING-CTONG 1103 trial (ClinicalTrials.gov identifier: NCT01407822), which explored the safety and efficacy of erlotinib compared with gemcitabine plus cisplatin (GC chemotherapy) as a neoadjuvant treatment in patients with stage IIIA-N2 NSCLC with EGFR mutations in exon 19 or 21.^[21–22]^ Their results showed that preoperative neoadjuvant targeted therapy could improve the complete resection rate and removal of micrometastases in the blood of patients with stage II–IIIA (N1-N2) NSCLC.

Gastric metastasis from lung cancer is relatively rare. Indeed, its frequency ranged from 0.19% to 5.1%.^[23–24]^ The most common sites of extrapulmonary spread include the liver (35%), bones (25%), adrenals (22%), kidneys (10%–15%), and heart pericardium (20%).^[25]^ However, the reported incidence of gastric metastasis from LUAD post-mortem is relatively high, ranging from 4.7% to 14%.^[26]^ Hence, this report describes a case of osimertinib resistance due to SCLC transformation and the gastric metastasis, the transformation from primary LUAD to SCLC, their features, prognosis and optimal treatment strategy after gastric metastasis were discussed.

## 2 Case report

**Figure 1:**
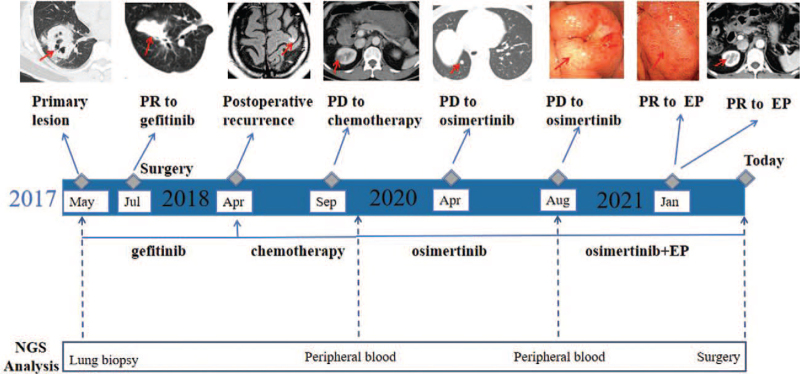
Timeline of the clinical course of the patient. Arrows indicate the link between the clinical event and the date of NGS time point.

**Figure 2:**
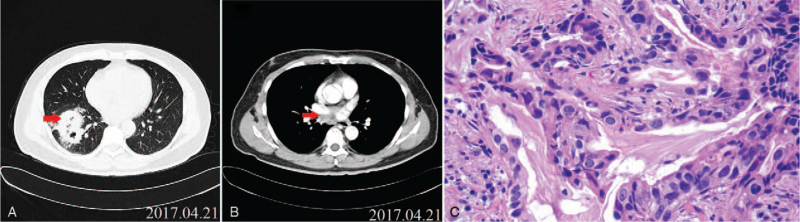
Computed tomography (CT) scan. (A) Chest scan indicating the presence of a nodule of 5.2 × 5.1 cm in the right lower lobe of the lung. (B) Subcarinal lymph node metastasis. (C) Lung specimen showing cancer cell infiltration (hematoxylin and eosin staining, 40x magnification).

**Figure 3:**
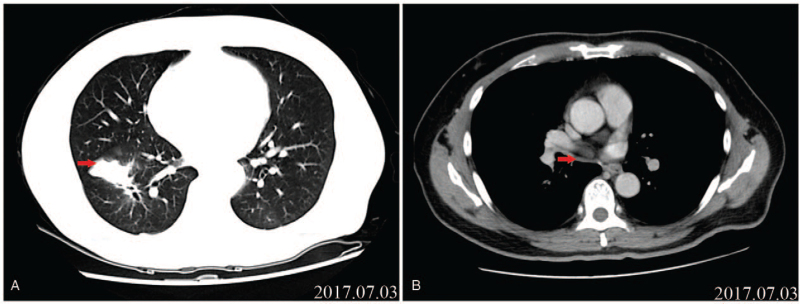
Computed tomography (CT) scan. (A) Chest scan indicating the presence of a nodule of 3.1 × 2.4 cm in the right lower lobe indicating the decrease in its mass after the use of gefitinib as neoadjuvant treatment. (B) Subcarinal lymph node metastasis disappearance after the use of gefitinib as neoadjuvant treatment.

A 58-year-old male Chinese patient, with no smoking history (only passive smoking), was admitted to the hospital because of cough and expectoration (Fig. 1). Computed tomography (CT) of the chest and abdomen revealed the presence of a primary tumor (with the longest diameter measuring 52 mm) in the right lower lobe (Fig. 2A) and subcarinal lymph node metastasis (Fig. 2B). A lung biopsy revealed the presence of adenocarcinoma cells (Fig. 2C). The clinical tumor stage was evaluated as T3N2M0 (stage IIIB). The potential presence of EGFR mutations was evaluated using a biopsy specimen, and the results revealed an exon 19 deletion in the EGFR gene. Hence, the patient was subjected to neoadjuvant therapy with the first-generation EGFR-TKI gefitinib (0.25 g). After 2 months (July 2017), a chest CT scan revealed a reduction in the right lower lobe mass (with the longest diameter measuring 31 mm) (Fig. 3A). Right lung middle lower lobe resection and mediastinal lymph node dissection were performed (Fig. 3B) (July 2017). The postoperative pathologic diagnosis was adenocarcinoma, and staging was pT2aN0M0 (stage IB). Subsequently, the patient was subjected to 4 cycles of chemotherapy composed of pemetrexed 800 mg (500 mg/m^2^ intravenously injected on day 1, once every 3 weeks) combined with Nida's platinum 60 mg (80 mg/m^2^ intravenously injected on day 1–2, once every 3 weeks). The patient achieved complete response.

After 6 months (April 2018), the patient complained of headaches, and brain magnetic resonance imaging revealed disease progression, with 3 lesions in the left parietal lobe, temporal lobe, and right frontal lobe. The patient continued to be treated with the first-generation EGFR-TKI gefitinib (0.25 g), achieving a partial response after 2 months, and all lesions were treated with gamma knife radiosurgery at a dose of 16 Gy (measuring curve of 50%).

**Figure 4:**
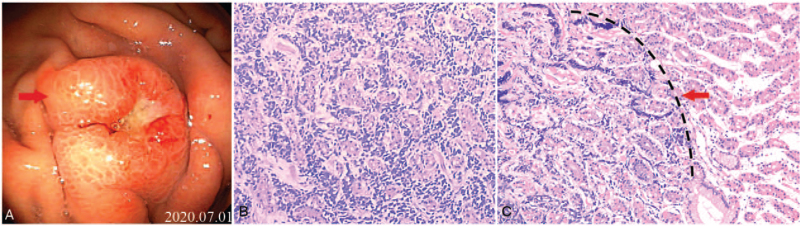
(A) Gastroscopy revealing a protruding lesion in the greater curvature of the stomach measuring 0.6 × 0.4 cm, with mucosa erosion and hyperemia. (B) Cancer cell infiltration. (C) Gastric specimen showing clear boundaries between the cancer tissue and normal gastric gland. (hematoxylin and eosin staining 40x magnification).

Five months after gefitinib (0.25 g) treatment (Sep 2018), the patient showed disease progression with a metastatic lesion in the right kidney. Thus, the therapy was subsequently replaced with standard pemetrexed 800 mg (500 mg/m^2^ intravenously injected on day 1, once every 3 weeks) combined with Nida's platinum 60 mg (80 mg/m^2^ intravenously injected on day 1–2, once every 3 weeks) (PP regime). The patient experienced loss of appetite and general weakness after 3 cycles, and the disease progressed further, involving the left kidney with another metastasis (November 2018). Therefore, the treatment was adjusted according to a personalized therapy, using bevacizumab 500 mg (7.5 mg/kg intravenously injected on day 1, once every 3 weeks) combined with pemetrexed 800 mg (500 mg/m^2^ intravenously injected on day 1, once every 3 weeks). The patient achieved stable disease after 1 cycle. However, the patient was not satisfied with the curative effect and was asked to change the therapy. Thus, the treatment was adjusted 1 more time to enhance the curative effect, and bevacizumab 500 mg (7.5 mg/kg intravenously injected at day 1, once every 3 weeks) combined with paclitaxel liposome 240 mg (135 mg/m^2^ intravenously injected at day 1, once every 3 weeks) was used. Blood samples were collected, and molecular testing of EGFR by amplification refractory mutation system-polymerase chain reaction detected an EGFR T790 M mutation. Therefore, the patient was treated with osimertinib (80 mg), and disease progression was observed after 1 and 3 months (April 2020). Computed tomography (CT) of the chest and abdomen revealed a secondary tumor (with the longest diameter measuring 12 mm) in the right upper lobe. Blood samples were collected again and subjected to next-generation sequencing, which revealed the presence of an EGFR T790 M mutation and the original exon 19 deletion. Subsequently, the patient was treated with hypofractionated radiotherapy in the right upper lobe (50 Gy/5 Gy/10 f) and continued osimertinib treatment. Unfortunately, CT performed after 2 months (June 2020) revealed disease progression due to an increase in the number of nodules in the right upper lobe posterior segment and right kidney. Magnetic resonance imaging revealed the enlargement of the lesions in the posterior horn of left lateral ventricle. The patient was subsequently treated with hypofractionated radiotherapy in the posterior horn of the left lateral ventricle (44 Gy/4 Gy/11 f, 3 Gy/1 f) and in the right upper lobe posterior segment (50 Gy/5 Gy/10 f). After radiotherapy, he developed a stomach, and gastroscopy with biopsy showed a small cell carcinoma in the gastric body derived from the lung (Fig. 4A and Fig. 4B). Blood samples were collected again and NGS detected an exon 19 deletion in the EGFR and T790 M mutations. Pathological examination after tissue staining showed that the metastatic tumor cells were localized in the submucosa of the stomach (Fig. 4C). Therefore, the patient was subjected to a standard treatment with etoposide 130 mg (100 mg/m^2^ intravenously injected on day 1–3, once every 3 weeks) and 45 mg of platinum (30 mg/m^2^ intravenously injected on day 1, once every 3 weeks) (EP) chemotherapy and maintenance therapy with osimertinib. Finally, the patient achieved a partial response after 4 cycles. The lesions in the left kidney and right kidney before chemotherapy are shown in Figure 6A and 6B. The left and right kidney lesions achieved a partial response after 4 cycles of treatment (Fig. 6D and Fig. 6E). Gastroscopy revealed that the gastric lesion had disappeared (Fig. 6C), but the biopsy of the mucosa still showed signs of a malignant tumor (Fig. 6F). Brain and lung lesions showed a stable disease state. In addition, the patient showed disease progression and an enlarged lesion in the stomach after the termination of chemotherapy. Surgery “partial gastrectomy” was performed. The potential presence of an exon 19 deletion in EGFR and TP53 mutations was evaluated using gastric postoperative specimens and blood samples.

## 3 Discussion

**Figure 5:**
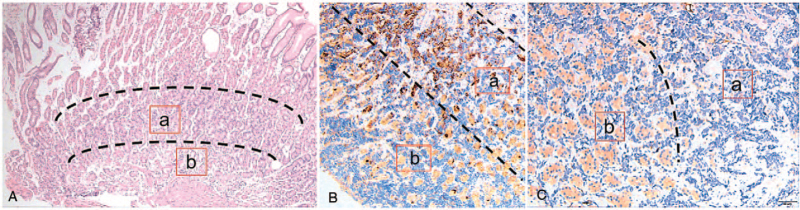
(A) Carcinoma cell infiltration into the submucosa of the stomach (hematoxylin and eosin staining, 40x magnification). (B) Representative image of CgA staining in tumor cells. (C) Representative image of CK7 staining in tumor cells. 200x magnification. a: near the mucosa. b: near the muscle.

**Figure 6:**
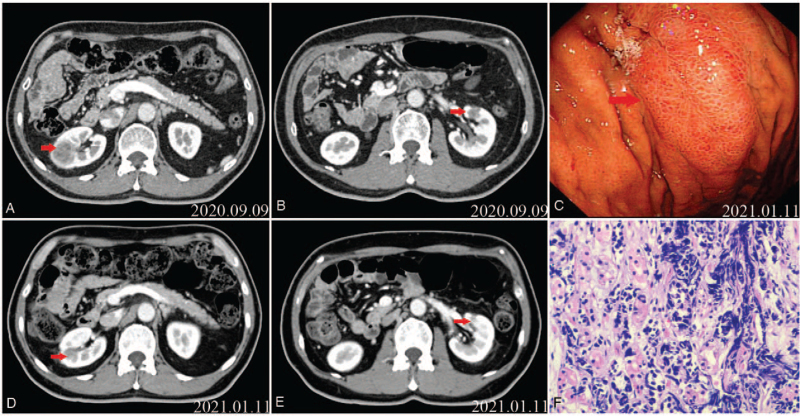
Computed tomography (CT) scan. (A) The nodule in the right kidney before EP chemotherapy. (B) The nodule in the left kidney before EP chemotherapy. (C) Gastroscopy revealing the disappearance of the protruding lesion in the greater curvature of the stomach. (D) The nodule in the right kidney after EP chemotherapy. (E) The nodule in the left kidney after EP chemotherapy. (F) Gastric specimen showing carcinoma cell infiltration after EP chemotherapy.

Metastatic disease involving the stomach is an unusual and difficult clinical problem. McNeer and Pack, in 1180 autopsies of patients with carcinoma, found that 0.7% of patients developed gastric metastases. A review of 1010 autopsies of patients with cancer revealed 17 cases of gastric metastases (an incidence of 1.7%), with breast cancer, lung cancer, and melanoma being the most frequent primaries.^[27]^ Gastric metastasis from primary lung carcinoma is rare. Squamous cell carcinoma (SCC) cells are the most common type of gastric metastasis.^[28]^ However, gastric metastasis from primary LUAD is unusual. The cases of gastric metastasis from primary LUAD cancer previously reported in the literature are summarized in Table 1. According to these previous cases, only four had a single metastasis in the stomach. Our study showed that a metastatic tumor was located above the body of the stomach. Esophagogastroduodenoscopy showed that most cases of metastasis present as submucosal tumors with mucosal folds up and modest ulcerations towards the top are often referred to as volcano-like ulcers. In general, tumors with these characteristics are regarded as hematogenous or lymphatic metastatic lesions, but this aspect has not been clarified in previous reports. The occurrence of gastric metastasis from LUAD has been increasing, although the cause is unclear. Two hypotheses were postulated: First, the sputum containing cancer cells is swallowed into the digestive tract, especially in smokers who are more susceptible to gastric mucosal damage.^[43]^ Thus, Helicobacter pylori infection and/or ulcers may be associated with gastric metastasis. Second, some cytokines may affect the organ specificity of blood metastasis.^[44]^ Our hypothesis is that our case was a hematogenous metastasis with a severe vascular but not lymphatic invasion, as histologically detected around the tumor. In addition, transformation to SCLC in patients with EGFR-sensitive mutations is not rare. In a study of 103 patients with T790 M positive mutation after the first generation of TKI resistance who were treated with osimertinib showed that the main mutations after osimertinib resistance were EGFR abnormality (25%), MET amplification (16%), TP53 mutation (8%), KRAS mutation (4%), RET fusion (4%), HER2 amplification (4%), and RB1 amplification (6.25%).^[45]^ RB1 mutation indicates the transformation from LUAD to SCLC. In our case, gastroscopy with biopsy indicated tumor cell infiltration, and immunohistochemistry confirmed that the small-cell cancer originated from the lung. Immunohistochemical staining for CD56, TTF-1, CgA, and CEA was positive, while EMA, CD3, and CD68 were negative, confirming the diagnosis of gastric small-cell cancer from LUAD. However, the patient underwent pathological changes after or before gastric metastasis from the lung. We hypothesized that gastric metastasis developed after the transformation. First, immunohistochemical staining showed that the tumor cells near the submucosa were significantly larger than those near the muscle layer (Fig. 5A). Furthermore, CgA expression was more remarkable in the submucosa and less in the muscular layer (Fig. 5B). In contrast, CK7 expression was more remarkable near the muscular layer and less in the submucosa (Fig. 5C). This suggests that the small cell transformation occurred before the invasion. Second, the lesion of the kidney continued to increase after osimertinib treatment alone. The lesions in the kidney inversely resulted in a partial response using EP and maintenance treatment with osimertinib (Fig. 6D and 6E).

**Table 1:** Reported cases of gastric metastasis from primary lung adenocarcinoma published in the literature.

			Smoking	Synchronous or	Time span (months)	Single or	Gastric	Clinical	Endoscopicc
No.	Author	Age/sex	history	metachronous		multiple organ	location	presentation	findings
1	Fushi Wei ^[29]^	60/M	NM	Metachronous	36	Multiple	Fundus	Abdominal distension	Not performed
2	Yuyan Wang ^[30]^	71/M	Y	Metachronous	>4	Single	Fundus	Fecal occult blood positive	SMT with ulcer
3	Qingyuan Huang ^[31]^	61/F	N	Metachronous	4	Single	Cardia	Epigastric discomfort	Not performed
4	Min Hee Lee ^[32]^	77/M	NM	Synchronous	0	NM	Antrum	Abdominal pain	Ulcer
5	Ryota Okazaki ^[33]^	68M	Y	Synchronous	0	Multiple	Body	Abdominal pain	SMT with ulcer
6	Engin Altintas ^[34]^	55M	NM	Metachronous	11	Multiple	Body	Melena	Ulcer
7	Xueqin Duan ^[35]^	41F	N	Synchronous	0	Multiple	Fundus	Abdominal distension	Ulcer
8	Yenmin Huang ^[36]^	41F	Y	Synchronous	0	Multiple	Body	Abdominal pain, Abdominal distension	Not performed
9	Michael Del Rosario ^[37]^	77F	N	Synchronous	0	NM	Body	NM	Ulcer
10	Yong Il Kim ^[23]^	71M	Y	Synchronous	0	Multiple	Body	‘Anemia	SMT with ulcer
11	Miyazaki, J. ^[38]^	54M	Y	Synchronous	0	Multiple	Antrum	Abdominal pain, Anemia	SMT with ulcer
12	Takayuki Jujo ^[39]^	73M	NM	Synchronous	0	Multiple	Body	None	SMT with ulcer
13	Punit Sharma ^[40]^	59M	N	Synchronous	0	Single	Body	None	Not performed
14	Charlotte Bouzbib ^[41]^	64M	Y	Synchronous	0	Single	Fundus	Acute anemia	SMT with ulcer
15	Stamatis Katsenos ^[42]^	61M	Y	Synchronous	0	Multiple	Body	Melena	SMT with ulcer
16	Our case	58M	N	Metachronous	39	Multiple	Body	Abdominal pain,	SMT with ulcer

F = female, M = male, N = no, NM = not mentioned, SMT = submucosal tumor, Y = yes. Time interval between the diagnosis of primary lung cancer and gastric metastasis.

In addition, at the time of progression to osimertinib, due to phenotypic transformation, our patient received standard platinum–etoposide chemotherapy and maintenance treatment with osimertinib, achieving clinical benefit. The identification of biomolecular mediators of treatment-dependent SCLC transformation represents a fundamental goal to develop therapeutic interventions. Current evidence supports TP53 and RB1 mutations as potential predictors of phenotypic switch in EGFR-mutated NSCLC.^[46]^ Moreover, a rapid increase in the serum levels of neuron-specific enolase (NSE), together with a poor response to EGFR-TKIs, usually indicates a transformation from adenocarcinoma to SCLC. In our case, TP53 mutations were detected by gastric tissue NGS analysis after osimertinib treatment, suggesting that the patient may have developed SCLC in the gastric mucosa. In conclusion, this report describes a patient with EGFR-mutant NSCLC small-cell type transformation and gastric metastasis after osimertinib treatment, suggesting the necessity of another biopsy because of the non-classical metastasis in the stomach. Another biopsy of the primary tumor could help us judge the sequence of transformation to SCLC and metastasis and initiate appropriate treatment. Although there is less occurrence of lung cancer with gastric metastasis, attention should be paid when gastrointestinal symptoms occur in patients with lung cancer. Timely detection of transformation to SCLC and initiation of appropriate treatment could improve the quality of life and prolong survival. Chemotherapy combined with EGFR-TKIs should be the appropriate treatment for these patients with SCLC transformation.

## Author contributions

**Methodology:** Lei Xia.

**Resources:** Zhenzhou Yang, Jing Liu, Yuan Peng, YuSheng Huang.

**Writing – original draft:** Jing Liu.

**Writing – review & editing:** Jing Liu.
